# Supplementary figures and images for: CASK and FARP localize two classes of post-synaptic ACh receptors thereby promoting cholinergic transmission
Source: PLoS Genet. 2022 Oct 24;18(10):e1010211. doi: 10.1371/journal.pgen.1010211 (PMC9632837; doi:10.1371/journal.pgen.1010211)

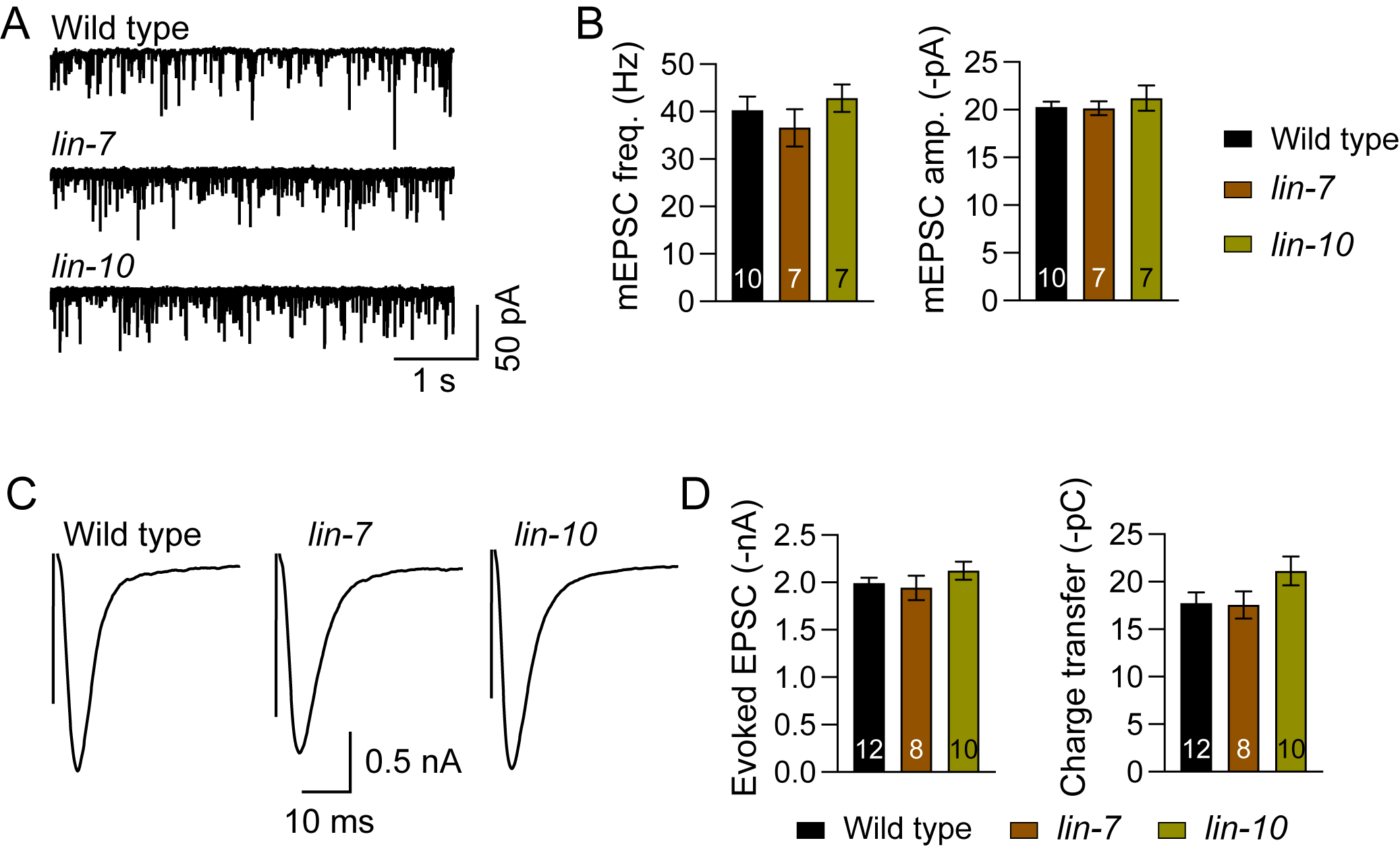

Supplement: S1 Fig — mEPSCs and evoked EPSCs were recorded in lin-7 and lin-10 mutants. Representative traces of mEPSCs and evoked EPSCs (A, C), and mean mEPSC frequency and amplitude, and evoked EPSC amplitude and charge transfer are shown. Data are mean ± SEM. The number of worms analyzed for each genotype is indicated in the bar. (TIF) [file pgen.1010211.s001.tif]

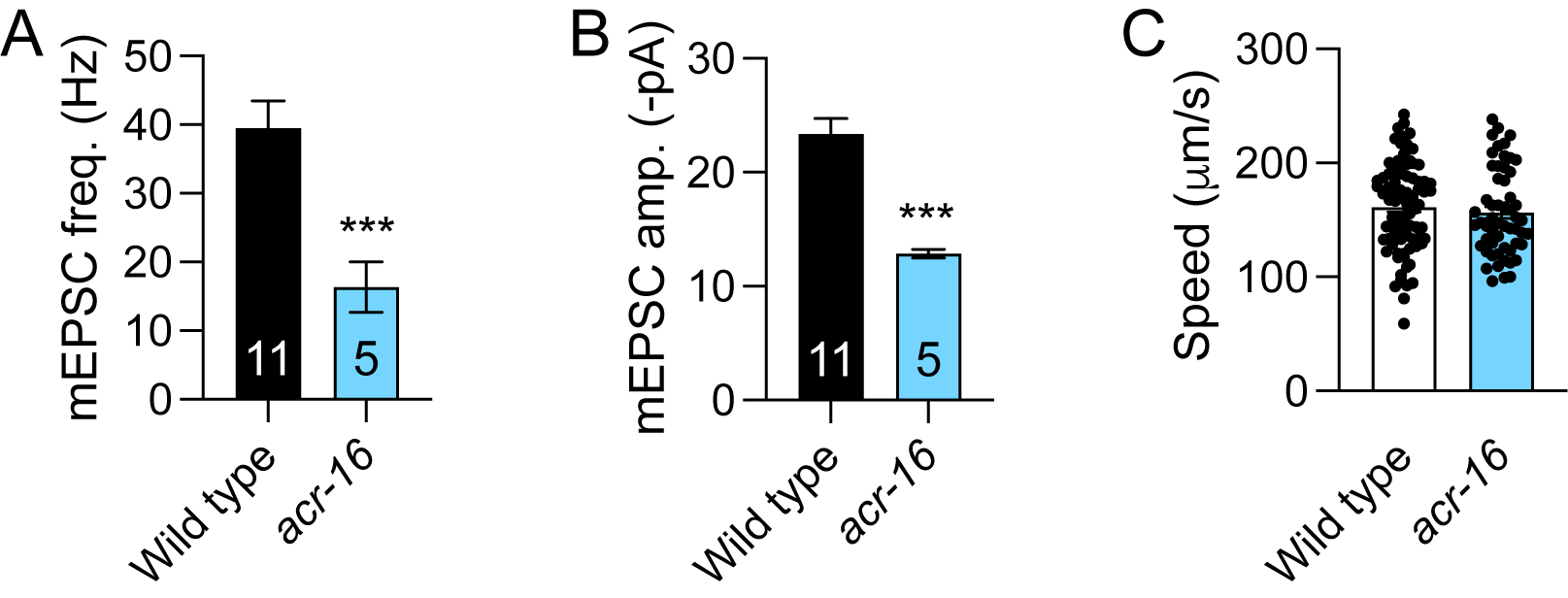

Supplement: S2 Fig — (A, B) Quantitation of mEPSC frequency and amplitude in wild type and acr-16 mutants. (C) Despite the severe defects in mEPSCs, acr-16 mutants exhibit normal locomotion speed. Data are mean ± SEM (***, p < 0.001 when compared to wild type; student’s t-test). The number of worms analyzed for each genotype is indicated in the bar. (TIF) [file pgen.1010211.s002.tif]

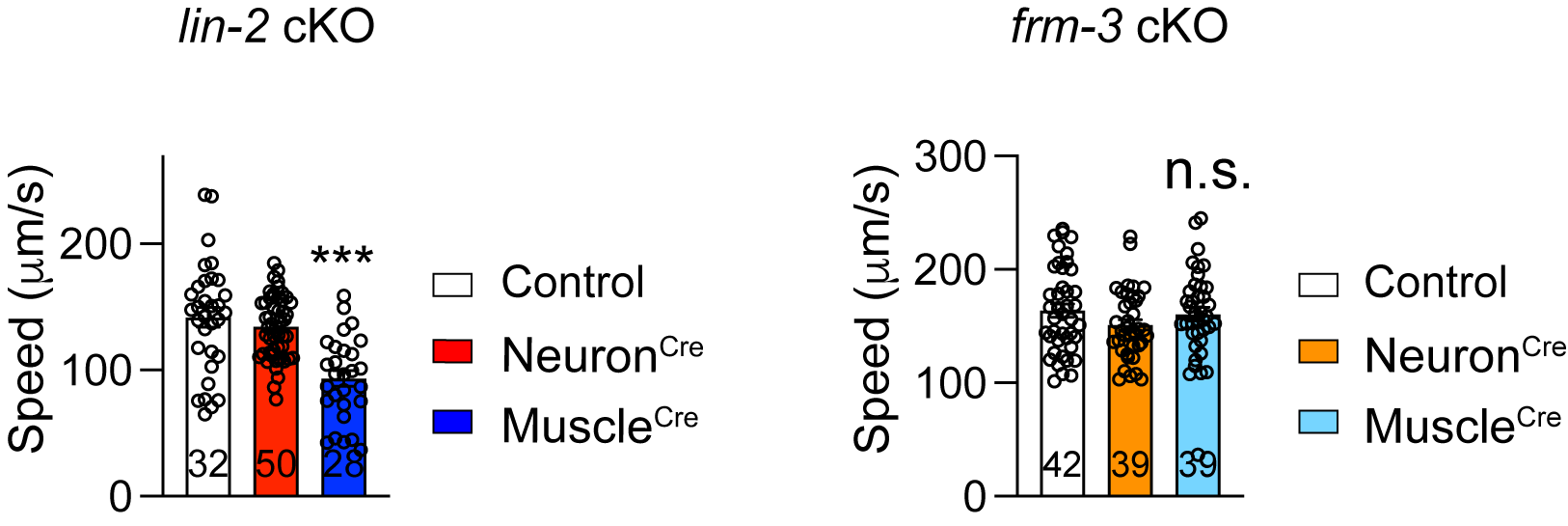

Supplement: S3 Fig — Locomotion was analyzed in two independent experiments in two days. The speed was decreased in lin-2 MuscleCre mutants but unchanged in frm-3 NeuronCre and MuscleCre mutants. Data are mean ± SEM (***, p < 0.001 when compared to Control animals; n.s., non-significant; one-way ANOVA). The number of worms analyzed for each genotype is indicated in the bar. (TIF) [file pgen.1010211.s003.tif]

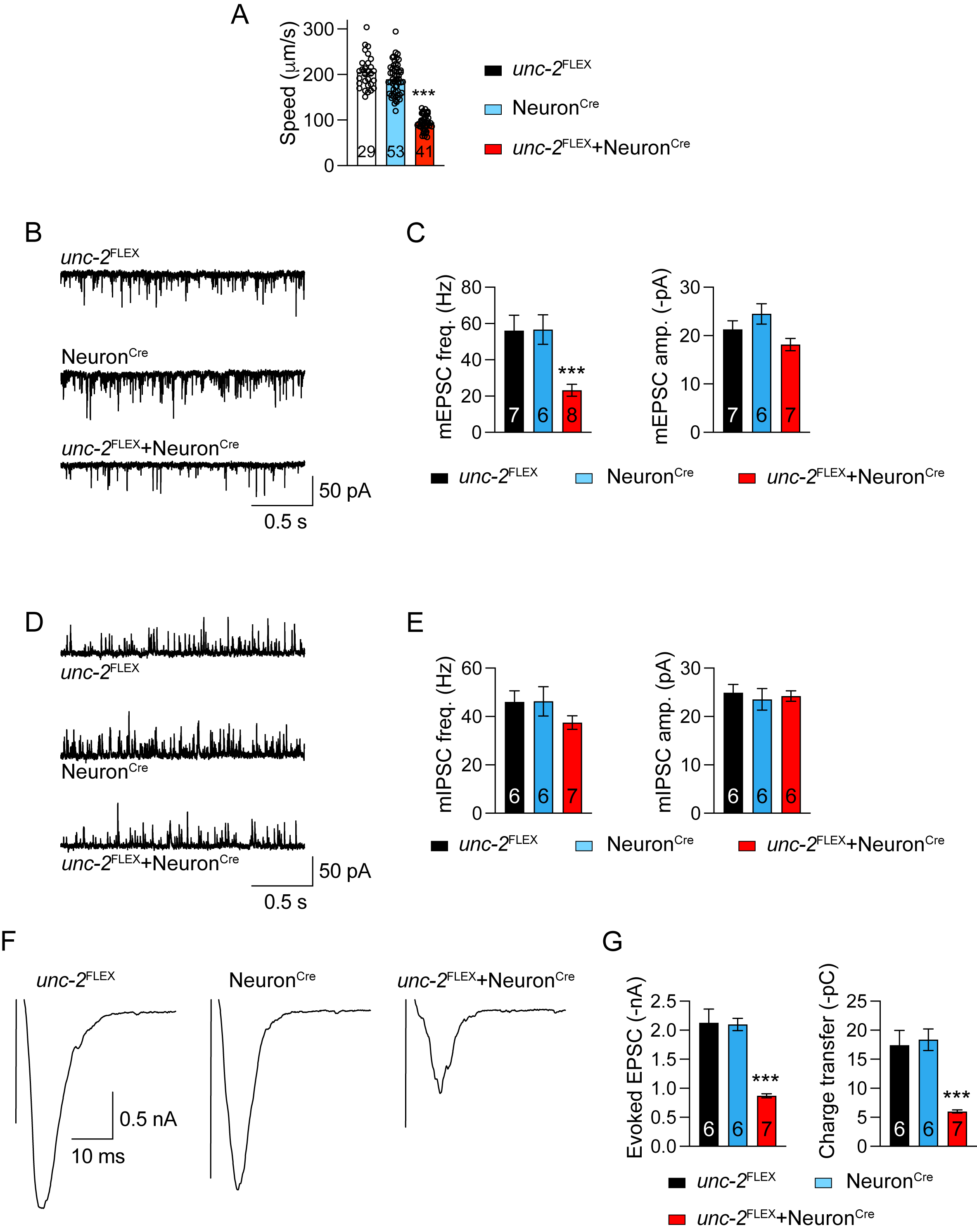

Supplement: S4 Fig — (A) Averaged speed in unc-2FLEX(nu657) mutants, wild type animals expressed NeuronCre, and unc-2FLEX(nu657) expressed NeuronCre. (B, C) Example traces of mEPSCs and summary of mEPSC frequency and amplitude from the same genotypes in A. (D, E) mIPSC traces and quantification of mIPSC frequency and amplitude. (F, G) Evoked EPSC traces and quantification of EPSC amplitude and charge transfer. Data are mean ± SEM (***, p < 0.001 when compared to unc-2FLEX(nu657); one-way ANOVA). The number of worms analyzed for each genotype is indicated in the bar. (TIF) [file pgen.1010211.s004.tif]

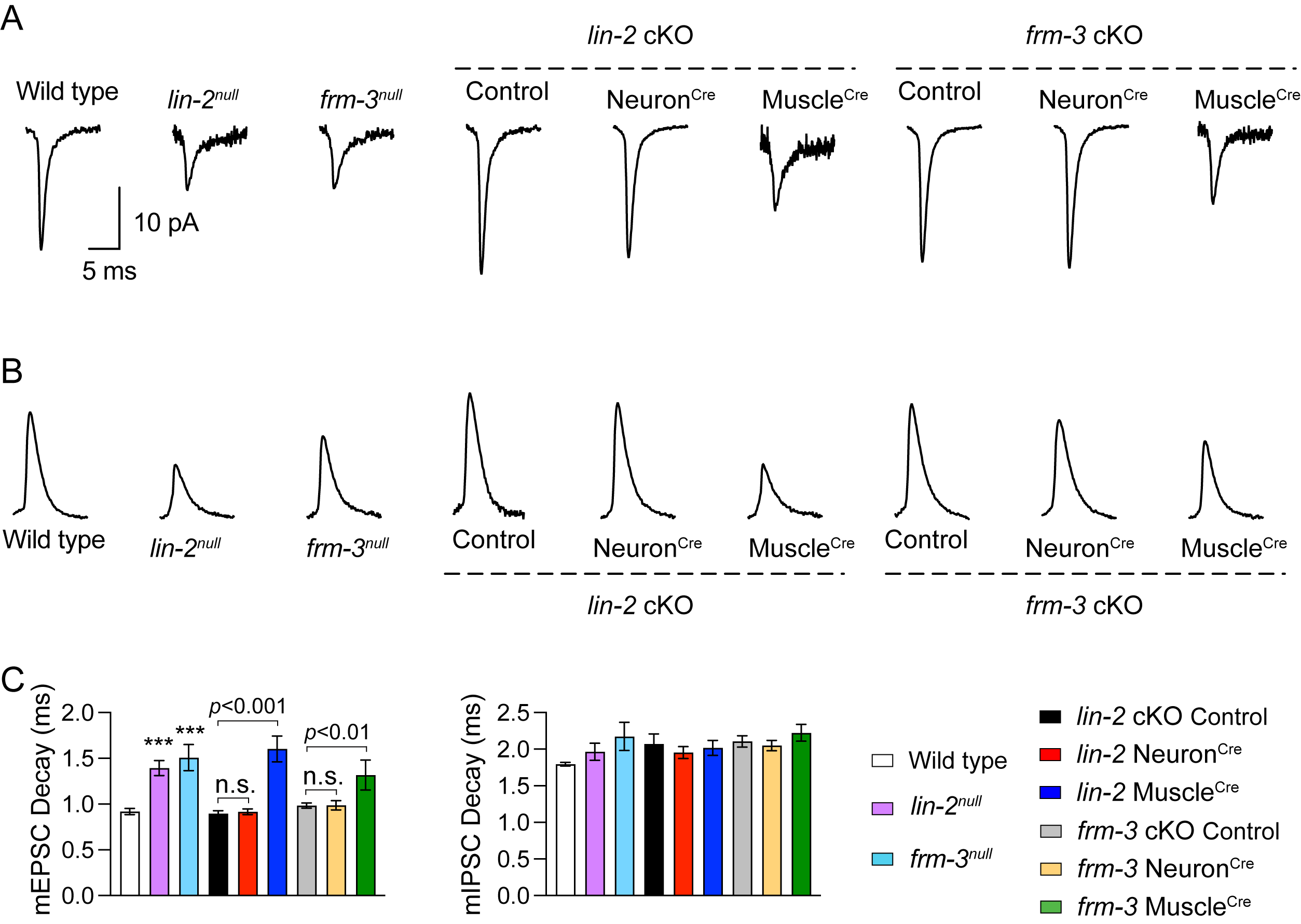

Supplement: S5 Fig — (A, B) Example of averaged mEPSCs and mIPSCs. Each current was averaged by all mini events in a single mEPSC or mIPSC trace (15 sec). (C) Quantification of the decay of the averaged mEPSCs and mIPSCs. Data are mean ± SEM (***, p < 0.001 when compared to wild type; n.s., non-significant; one-way ANOVA). The number of worms analyzed for each genotype is indicated under each box. (TIF) [file pgen.1010211.s005.tif]

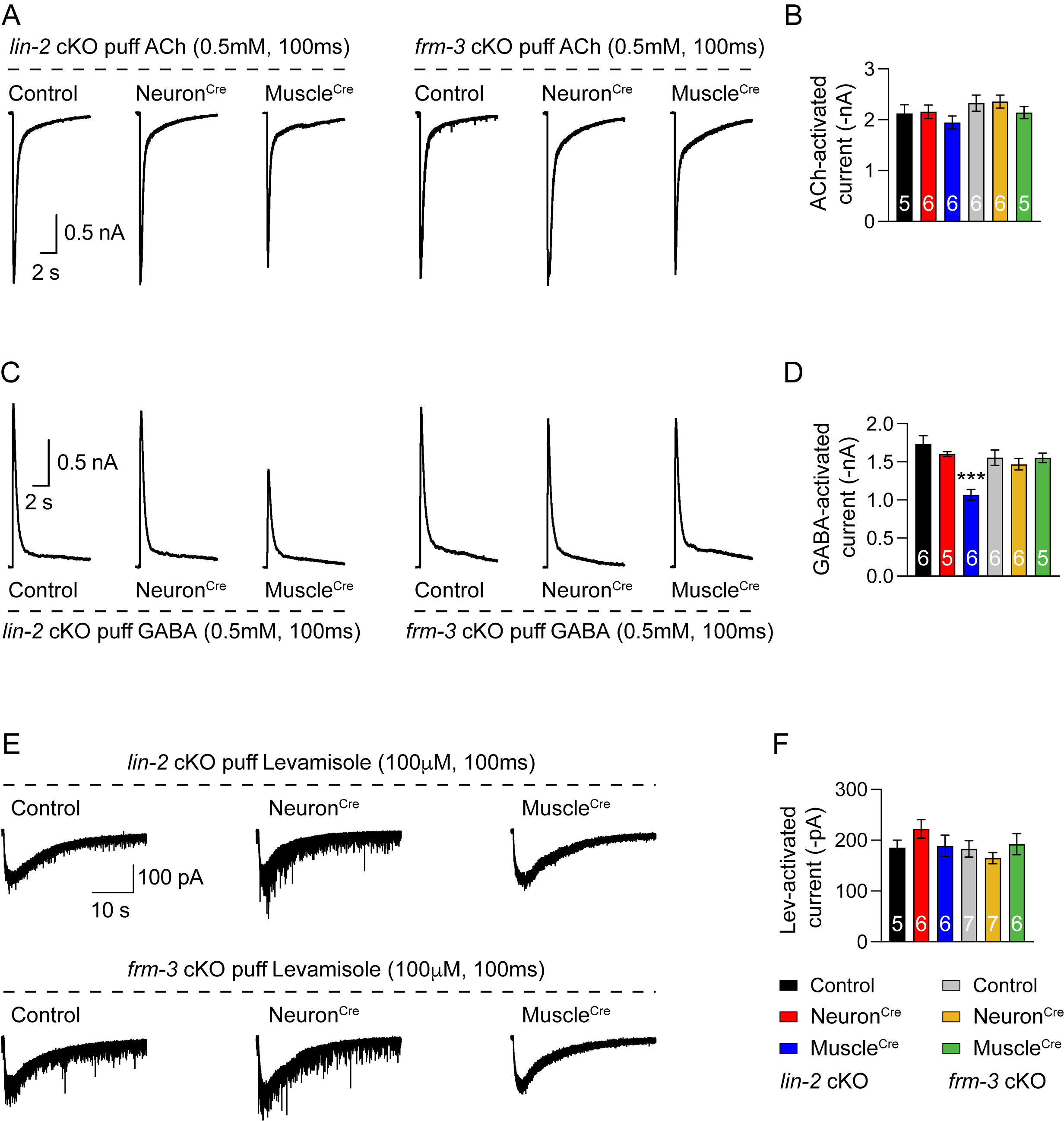

Supplement: S6 Fig — (A, C, E) Representative traces of ACh-, GABA-, and levamisole-activated currents in neuron and muscle conditional knockout mutants of lin-2 and frm-3. (B, D, F) Quantification of current amplitude. Data are mean ± SEM (***, p < 0.001 when compared to control; one-way ANOVA). The number of worms analyzed for each genotype is indicated in the bar. (TIF) [file pgen.1010211.s006.tif]
