# Supplementary material for: CASK and FARP localize two classes of post-synaptic ACh receptors thereby promoting cholinergic transmission
Source: PLoS Genet. 2022 Oct 24;18(10):e1010211. doi: 10.1371/journal.pgen.1010211 (PMC9632837; doi:10.1371/journal.pgen.1010211)
Supplement: S1 Table — Data are presented as mean ± SEM. (PDF) [file pgen.1010211.s007.pdf]

Table S1. Summary of the locomotion speed data in this study.

	Speed (μm/s)	
	Experiment 1	Experiment 2
Wild type	162 ± 5.87	165 ± 5.83
lin-2(e1309)	117 ± 13	119 ± 9.87
frm-3(gk585)	121 ± 4.31	121 ± 6.4
lin-2 (null)	117 ± 5.94	121 ± 4.75
frm-3 (null)	124 ± 6.71	126 ± 6.25
lin-2(e1309);frm-3(gk585)	107 ± 4.48	109 ± 5.1
lin-2(nu473)	142 ± 7.83	n/a
lin-2(nu473) ;NeuronCre	135 ± 3.54	n/a
lin-2(nu473) ;MuscleCre	93.3 ± 6.27	n/a
frm-3(nu751)	164 ± 5.91	n/a
frm-3(nu751) ;NeuronCre	151 ± 4.82	n/a
frm-3(nu751) ;MuscleCre	161 ± 6.24	n/a
Wild type	161 ± 4.32	n/a
acr-16	157 ± 5.05	n/a
unc-2(nu657)	205 ± 6.71	n/a
NeuronCre	190 ± 4.78	n/a
unc-2(nu657) ;NeuronCre	95.1 ± 2.71	n/a

Data are presented as the mean +/- SEM.
