# Supplementary material for: CASK and FARP localize two classes of post-synaptic ACh receptors thereby promoting cholinergic transmission
Source: PLoS Genet. 2022 Oct 24;18(10):e1010211. doi: 10.1371/journal.pgen.1010211 (PMC9632837; doi:10.1371/journal.pgen.1010211)
Supplement: S2 Table — Data are presented as mean ± SEM. (PDF) [file pgen.1010211.s008.pdf]

Table S2. Summary of the spontaneous and evoked EPSC data in this study.

	mEPSC		mIPSC		Evoked EPSC	
	Frequency (Hz)	Amplitude (-pA)	Frequency (Hz)	Amplitude (pA)	Amplitude (pA)	Charge (-pC)
Wild type	43.5 ± 4.3	23.9 ± 1.2	43.5 ± 4.3	23.8 ± 2.2	2.3 ± 0.2	20.6 ± 2.1
lin-2(e1309)	27 ± 4.2	15.4 ± 0.4	27 ± 4.2	19.4 ± 1.1	0.49 ± 0.07	5.5 ± 0.7
frm-3(gk585)	8.1 ± 1.6	12.1 ± 0.5	8.05 ± 1.6	17.3 ± 0.5	0.32 ± 0.03	3.1 ± 0.5
lin-2(e1309);frm-3(gk585)	6.1 ± 1.7	13.3 ± 0.9	6.1 ± 1.7	16.3 ± 0.3	0.24 ± 0.03	2.78 ± 0.37
lin-2 (null)	2.1 ± 0.36	12 ± 0.2	2.1 ± 0.4	14.7 ± 0.4	0.2 ± 0.06	2 ± 0.74
frm-3 (null)	3.7 ± 0.71	12.6 ± 0.35	3.7 ± 0.7	14.7 ± 0.4	0.27 ± 0.04	2.8 ± 0.42
lin-2(nu473)	50.1 ± 6.6	21.2 ± 0.9	41 ± 6.7	21 ± 1.7	2.1 ± 0.32	20.3 ± 3.2
lin-2(nu473) ;NeuronCre	57.8 ± 5.2	23.6 ± 1.4	51.6 ± 6.7	22.6 ± 1.8	1.82 ± 0.2	16.7 ± 1.4
lin-2(nu473) ;MuscleCre	1.68 ± 0.4	13.1 ± 0.77	21 ± 5.7	12.8 ± 0.8	0.179 ± 0.03	2.35 ± 0.5
frm-3(nu751)	42.6 ± 3.3	20.8 ± 1.49	43.2 ± 4.1	21.1 ± 1.0	2.1 ± 0.1	18.1 ± 1.3
frm-3(nu751) ;NeuronCre	49.6 ± 5.9	22.4 ± 1.8	47 ± 4.4	23.7 ± 2.2	1.86 ± 0.2	15.8 ± 2.6
frm-3(nu751) ;MuscleCre	8.66 ± 3.1	12.2 ± 0.4	32.3 ± 6.4	14.2 ± 1.1	0.395 ± 0.03	3.8 ± 0.3
Wild type	41.3 ± 4.0	23.9 ± 1.8	43.4 ± 7.03	24.1 ± 3.1	2.26 ± 0.2	21.5 ± 3.2
∆SH3	1.97 ± 0.2	12.1 ± 0.5	28.9 ± 7.47	13.5 ± 1.0	0.28 ± 0.02	3.01 ± 0.4
∆PDZ	2.8 ± 0.4	12.8 ± 0.8	34.1 ± 7	14 ± 0.9	0.25 ± 0.03	2.65 ± 0.5
Wild type	40.3 ± 2.9	20.3 ± 0.5	n/a	n/a	1.99 ± 0.05	17.8 ± 1.1
lin-7	36.6 ± 3.9	20.2 ± 0.7	n/a	n/a	1.94 ± 0.13	17.6 ± 1.4
lin-10	42.8 ± 2.8	21.2 ± 1.3	n/a	n/a	2.12 ± 0.09	21.1 ± 1.5
Wild type	39.5 ± 3.9	23.4 ± 1.3	n/a	n/a	n/a	n/a
acr-16	16.4 ± 3.6	12.9 ± 0.3	n/a	n/a	n/a	n/a
unc-2(nu657)	56.1 ± 8.4	21.3 ± 1.8	46 ± 4.6	24.9 ± 1.7	2.13 ± 0.2	17.4 ± 2.5
NeuronCre	56.7 ± 8.1	24.5 ± 2.0	46.3 ± 6.0	23.6 ± 2.2	2.1 ± 0.1	18.4 ± 1.8
unc-2(nu657) ;NeuronCre	23.2 ± 3.2	18.1 ± 1.2	37.5 ± 2.7	24.2 ± 1.1	0.87 ± 0.03	5.9 ± 0.2

Data are presented as the mean +/- SEM.
