# Supplementary material for: CASK and FARP localize two classes of post-synaptic ACh receptors thereby promoting cholinergic transmission
Source: PLoS Genet. 2022 Oct 24;18(10):e1010211. doi: 10.1371/journal.pgen.1010211 (PMC9632837; doi:10.1371/journal.pgen.1010211)
Supplement: S3 Table — Data are presented as mean ± SEM. (PDF) [file pgen.1010211.s009.pdf]

Table S3. Summary of the imaging data in this study.

	ACR-16::RFP (peak, %WT)	UNC-29::RFP (peak, %WT)	UNC-57::mCherry (peak, %WT)	UNC-57::mCherry (density, %WT)
Wild type	1 ± 0.035	1 ± 0.03	1 ± 0.028	1 ± 0.022
lin-2 (null)	0.62 ± 0.016	0.75 ± 0.02	1.1 ± 0.056	1 ± 0.023
frm-3 (null)	0.59 ± 0.017	0.55 ± 0.016	1.1 ± 0.059	1 ± 0.019
lin-2;frm-3	0.6 ± 0.024	0.51 ± 0.013	n/a	n/a

Data are presented as the mean +/- SEM.
