# Supplementary material for: CASK and FARP localize two classes of post-synaptic ACh receptors thereby promoting cholinergic transmission
Source: PLoS Genet. 2022 Oct 24;18(10):e1010211. doi: 10.1371/journal.pgen.1010211 (PMC9632837; doi:10.1371/journal.pgen.1010211)
Supplement: S4 Table — Data are presented as mean ± SEM. (PDF) [file pgen.1010211.s010.pdf]

Table S4. Summary of ACh, Levamisole, and GABA-activated currents in this study.

	Puff ACh (-nA)	Puff Levamisole (-pA)	Puff GABA (-nA)
Wild type	1.52 ± 0.09	239 ± 14.8	1.31 ± 0.07
lin-2(null)	1.53 ± 0.11	207 ± 7.76	0.63 ± 0.03
frm-3(null)	1.49 ± 0.07	231 ± 25	1.19 ± 0.05
lin-2(nu473)	2.13 ± 0.17	185 ± 14.6	1.74 ± 0.10
lin-2(nu473) ;NeuronCre	2.16 ± 0.13	222 ± 18.2	1.6 ± 0.03
lin-2(nu473) ;MuscleCre	1.95 ± 0.12	189 ± 21.2	1.07 ± 0.07
frm-3(nu751)	2.33 ± 0.16	183 ± 16.1	1.56 ± 0.10
frm-3(nu751) ;NeuronCre	2.36 ± 0.13	164 ± 11	1.47 ± 0.07
frm-3(nu751) ;MuscleCre	2.14 ± 0.12	192 ± 20.8	1.55 ± 0.06

Data are presented as the mean +/- SEM.
