# Supplementary material for: CASK and FARP localize two classes of post-synaptic ACh receptors thereby promoting cholinergic transmission
Source: PLoS Genet. 2022 Oct 24;18(10):e1010211. doi: 10.1371/journal.pgen.1010211 (PMC9632837; doi:10.1371/journal.pgen.1010211)
Supplement: S5 Table — Data are presented as mean ± SEM. (PDF) [file pgen.1010211.s011.pdf]

Table S5. Summary of mini decay.

	mEPSC decay (ms)	mIPSC decay (ms)
Wild type	0.92 ± 0.03	1.79 ± 0.0252
lin-2 (null)	1.39 ± 0.08	1.97 ± 0.115
frm-3 (null)	1.51 ± 0.14	2.17 ± 0.193
lin-2(nu473)	0.89 ± 0.03	2.07 ± 0.14
lin-2(nu473) ;NeuronCre	0.92 ± 0.03	1.96 ± 0.080
lin-2(nu473) ;MuscleCre	1.6 ± 0.14	2.02 ± 0.10
frm-3(nu751)	0.98 ± 0.03	2.11 ± 0.07
frm-3(nu751) ;NeuronCre	0.98 ± 0.05	2.05 ± 0.07
frm-3(nu751) ;MuscleCre	1.32 ± 0.16	2.22 ± 0.11

Data are presented as the mean +/- SEM.
