# Supplementary material for: CASK and FARP localize two classes of post-synaptic ACh receptors thereby promoting cholinergic transmission
Source: PLoS Genet. 2022 Oct 24;18(10):e1010211. doi: 10.1371/journal.pgen.1010211 (PMC9632837; doi:10.1371/journal.pgen.1010211)
Supplement: Attachment — Submitted filename: Rebuttal.pdf [file pgen.1010211.s013.pdf]

**Overview:**

We are pleased that the reviewers found our results to be of interest. The reviews provide many helpful suggestions, which we believe have greatly improved the manuscript. In response to these comments, we added several new experiments. 1) We analyzed the locomotion speed, mEPSC and mIPSC decay kinetics, and agonist (ACh, levamisole, and GABA) activated currents in *lin-2* and *frm-3* conditional knockout mutants. 2) We evaluated the efficiency of the Cre recombinase in neurons by testing its effects in inactivating the *unc-2* gene. 3) We analyzed the mEPSCs in *acr-16* (encodes nicotine-sensitive receptors) mutants. The analysis showed that large decrease in mEPSC amplitude reduces mEPSC frequency. This helped us understand how the mEPSC frequency is decreased in *lin-2* and *frm-3* mutants. 4) We recorded mIPSCs in almost all mutants analyzed, including *lin-2* and *frm-3* null mutants, conditional knockout mutants, and *lin-2* ΔSH3 and ΔPDZ mutants. 5) We analyzed the locomotion speed in *acr-16* mutants. This allowed us to understand how worm movement is regulated by NMJ synaptic currents mediated by the two acetylcholine receptors. 6)We added a model figure illustrating how LIN-2/CASK and FRM-3/FRAP regulate cholinergic and GABAergic transmission. We did not analyze cholinergic transmission in the *lin-2;frm-3* double null mutants because the dramatic mEPSC frequency defects in *lin-2* and *frm-3* single mutants (> 90% reduced) makes it difficult to detect additive effects in double null mutants. In contrast, the results in the double hypomorphic mutants showed very clearly that LIN-2/CASK and FRM-3/FARP function in the same genetic pathway.

All three reviewers expressed concern about the proposed retrograde synaptic defects in *lin-2* and *frm-3*. We agree with these concerns and have deleted all discussion of potential retrograde pre-synaptic defects in these mutants.

We reorganized the manuscript incorporating the new results into the figures and text. Full responses to all reviewer comments are detailed below. We hope that the reviewers agree that these changes address their major concerns and that our manuscript is now acceptable for publication in the *PLOS Genetics*.

**Reviewer #1:** please see attached review

This study by Hu and colleagues aims to understand how LIN-2/Cask and FRM-3/FARP regulate cholinergic transmission. While the LIN-2/FRM-3 complex has been evaluated in GABA transmission, this study examines effects on cholinergic transmission for the first time. A highlight of the study is the tremendous range of approaches including behavioral analysis, extensive electrophysiology, traditional genetics, cell-specific knockouts, yeast two-hybrid and CRISPR editing.

The study takes two important mechanistic steps. 1) LIN-2 and FRM-3 are required to localize acetylcholine receptors to postsynaptic terminals. This provides a cell biological explanation for the cholinergic transmission defects in lin-2 and frm-3 mutants. 2) The SH3 domain of LIN-2 was found to bind ACR-16 and UNC-29 ACh receptor subunits, and the LIN-2 PDZ domain interacts with FRM-3. Results with CRISPR editing to remove specific domains in LIN-2 and functional outcomes were consistent with biochemical findings. Thus, the authors' results suggest that the FRM-3/LIN-2 complex binds UNC-16 and UNC-29 ACh receptors to regulate their localization and cholinergic transmission.

Below the reviewer raises several concerns with the goal of solidifying what is a potentially excellent study.

**Major Comments**

1) For Figures 1 and 2, rescue experiments for frm-3 and lin-2 single mutants, and analysis of lin-2 frm-3 double mutants using null alleles are needed to strengthen conclusions. Given that the authors argue LIN-2 and FRM-3 form a complex and function in the same pathway, multi-gene genetics needs to be done with null alleles to be conclusive. Regarding electrophysiology it seems reasonable to only perform rescues and double mutant analysis on a portion of the cholinergic transmission readouts.

As requested, we now provide imaging results showing that synaptic AChR levels are severely decreased in *lin-2* and *frm-3* null mutants, but are not further decreased in *lin-2;frm-3* double null mutants (Figure 4), indicating that LIN-2 and FRM-3 act in the same genetic pathway. We are unable to assess additivity for mEPSC frequency in *lin-2;frm-3* double null mutants because mEPSCs are nearly eliminated in both single mutants (Figure 2).

Instead of rescue data, we analyzed phenotypes of *frm-3* and *lin-2* neuron and muscle specific conditional knockouts using CRE inactivated alleles. We prefer the conditional knockout strategy because it avoids problems associated with transgenic rescue, e.g. over-expression artifacts, and contribution of different protein isoforms (e.g. *lin-2a*, *lin-2b*, *frm-3a*, and *frm-3b*) to rescuing activity. Our results clearly show that the loss of *lin-2* and *frm-3* in body wall muscles but not neurons lead to strong transmission defects, and provided clear mechanisms for how LIN-2 and FRM-3 function in cholinergic and GABAergic synapses. We hope that these data address the reviewer's concern.

2) LIN-2 and FRM-3 affect presynaptic and postsynaptic transmission. Isn't it important to test their cell-specific activity in not only postsynaptic muscles but also presynaptic cholinergic motor neurons? While the authors have used a pan-neuronal driver this is not necessarily specific enough and might not yield efficient CRE recombination in cholinergic motor neurons. This is an important experiment to shore up the trans-synaptic signaling model because LIN-2 and FRM-3 are expressed in cholinergic motor neurons.

To confirm that pan-neuronal CRE expression produces efficient recombination in ACh motor neurons, we show that the pan-neuronal CRE transgene (P*sbt-1*::CRE) expressed in a CRE-inactivated *unc-2(nu657* FLEX*)* mutant dramatically decreased locomotion speed and spontaneous and evoked EPSCs (Figure S4). These phenotypes are similar to those observed in *unc-2* mutants (Liu et al., 2018, Cell Reports):

Page 10:

"The unchanged synaptic transmission in the Neuron$^{Cre}$ mutants of *lin-2* and *frm-3* may arise from a failure of the Cre recombinase to inactivate *lin-2* and *frm-3* in ACh neurons. To address this concern, we asked if Cre recombinase expression could inactivate an essential pre-synaptic gene, *unc-2*. The *unc-2* gene encodes the P/Q-type $Ca^{2+}$ channel in *C. elegans*. Loss of *unc-2* function leads to strong decrease in both locomotion rate and $Ca^{2+}$-dependent neurotransmitter release (Tong et al., 2017; Liu et al., 2018). The *unc-2*(*nu657*) allele contains a STOP cassette in intron 17. The presence of CRE is expected to inactivate *unc-2*, leading to behaviour and transmission

defects. As shown in Figure S4, Neuron$^{Cre}$ in *unc-2 (nu657)* significantly decreased locomotion speed, mEPSC frequency, and evoked EPSC amplitude and charge transfer. These results are similar to those observed in *unc-2* mutants, demonstrating that the Cre recombinase is highly efficient in cholinergic motor neurons."

3) The authors do a nice job discussing trans-synaptic effects of MAGUKs, Mint and FARP. While the reviewer very much appreciates this, a question comes to mind. Why would frm-3 and lin-2 principally function postsynaptically in muscle but yield stronger effects on presynaptic transmission (mEPSC frequency) than postsynaptic transmission (EPSC amplitude)? Is this the case in the other examples the authors cite?

As recommended in review #2 (see major comment #2 below), we no longer comment on potential changes in pre-synaptic function in the muscle specific *frm-3* and *lin-2* knockouts. We agree that the mEPSC rate and Quantal Content phenotypes could be entirely explained by the decreased muscle ACh receptor levels in these mutants.

4) Is the evoked EPSC as strong with muscle specific knockouts as null alleles? This does not look to be the case. Could the authors clarify?

As requested, we now compare evoked EPSC defects observed in *lin-2* and *frm-3* muscle specific and constitutive null mutants. Similar evoked EPSC defects are seen in *lin-2* Muscle$^{Cre}$ mutants (87% decrease) and *lin-2* null mutants (90% decrease). In the original submission, evoked EPSCs in *frm-3* Muscle$^{Cre}$ mutants (61% decrease) were slightly larger than those in *frm-3* null mutants (88% decrease). To clarify this, we did additional recordings. These new data suggest that that the evoked EPSCs in *frm-3* Muscle$^{Cre}$ mutants (80% decrease) are similar to those in *frm-3* null mutants. These results have been incorporated into Figure 3 in the revised manuscript.

5) The use of CRISPR to delete SH3 and PDZ domains from LIN-2 is a very nice addition to the study, and complementary to yeast two-hybrid results. However, the reviewer urges some caution in interpreting the delta SH3 and delta PDZ CRISPR alleles. Are the authors sure that these deletion mutants do not destabilize the overall LIN-2 protein structure creating null alleles? Similarly, negative results in yeast two hybrid experiments might result from failure of construct expression.

The ΔSH3 and ΔPDZ mutants exhibit severe defects in cholinergic synaptic transmission (mEPSCs, evoked EPSCs) similar to the *lin-2* null mutants (Figure 2 and Figure 8). By contrast, mIPSC frequency was not significantly changed in these mutants, unlike the *lin-2* null mutant. These results provide evidence that LIN-2ΔSH3 and LIN-2ΔPDZ truncations are still functional, and the lack of SH3 or PDZ unlikely destabilize the overall LIN-2 structure. We have incorporated these results into the revised manuscript.

Page 17:

"The functional importance of the SH3 and PDZ domains was also evaluated in GABAergic synapses. The mIPSC amplitude in ΔSH3 and ΔPDZ mutants was decreased to a similar extent to that in *lin-2* null mutants (Figure 8G, H). However, the mIPSC frequency was not changed in the ΔSH3 and ΔPDZ mutants, unlike *lin-2* null mutants (Figure 2). These results suggest that the mutant LIN-2 proteins are expressed and retain some function."

Similarly, it is unlikely that decreased expression caused the negative results in yeast two hybrid experiments, because LIN-2ΔSH3 and LIN-2ΔPDZ exhibit interaction with both FRM-3 (Figure 7F) and ACR-16 (Figure 7B). Thus, both constructs were expressed in yeast.

**Minor Comments**

1) The reviewer was a bit puzzled. What is the role of lin-2 and frm-3 in motor neurons, if it is acting exclusively in muscle to affect both pre and postsynaptic cholinergic transmission? Is anything know about other roles for LIN-2 or FRM-3 in presynaptic neurons/terminals? Perhaps the authors might comment on this in the Discussion?

As recommended by reviewer #2 (see major comment #2 below), we no longer comment on potential pre-synaptic defects following muscle specific *frm-3* and *lin-2* knockout. We agree that the mEPSC rate and Quantal Content phenotypes could be entirely explained by the decreased muscle ACh receptor levels.

2) Wouldn't such large effects on cholinergic transmission, as observed in lin-2 and frm-3 null mutants, precipitate larger defects in locomotion? Perhaps another known control for reduced cholinergic transmission might be included in locomotor behavioral studies?

We thank the reviewer for this comment. This issue was not addressed in our original submission. The locomotion defects observed are entirely consistent with the muscle ACh receptor defects we observed in *lin-2* and *frm-3* null mutants. In both null mutants, we observed ~50% decrease in muscle ACR-16 and UNC-29 puncta intensity (Figure 4A-D). Locomotion rate is unaltered in *acr-16* null mutants (see Figure S2, and Babu Neuron 2011). By contrast, locomotion rate is dramatically reduced in *unc-29* mutants (Touroutine et al., 2005, Journal of Biological Chemistry). Thus, the relatively mild locomotion defects observed in *lin-2* and *frm-3* mutants are likely explained by the residual synaptic UNC-29 levels in these mutants. In response to this comment, we now explain these results more clearly in the revised text:

Page 8:

"Moreover, the severe defects in mEPSCs but normal locomotion speed in the *acr-16* mutants (Figure S2C) also explained the moderate decrease in locomotion in *lin-2* and *frm-3* mutants."

Page 20:

"Although post-synaptic currents are dramatically reduced, locomotion rates were only modestly reduced in *lin-2* and *frm-3* null mutants. This persistent locomotion behavior likely reflects the residual function of post-synaptic L-AChRs in these mutants."

3)The data in Figure 1F seems to be presented as individual animals. It would be helpful if the legend noted the number of independent experiments that were performed. The reviewer is concerned that reporting individual animals is not ideal compared to reporting the average locomotion speed from multiple experiments and then running statistics. The authors might consider reanalyzing this data. C. elegans is a model where large numbers of animals can be analyzed and data can be presented across independent experiments rather than using individual animals.

To confirm the repeatability of the data, the locomotion speed was measured in two independent experiments in two days (Experiment 1 and Experiment 2; Figure 1F). For each mutant, around 10-40 animals were analyzed in one experiment. Statistical significance was tested for each experiment. We have added the information in the legend of Figure 1.

Page 32:

"To measure locomotion speed, young adult animals were washed with a drop of PBS and then transferred to fresh NGM plates with no bacterial lawn (30 worms per plate). Worm movement recordings (under room temperature 22°C) were started 10 min after the worms were transferred. A 2 min digital video of each plate was captured at 3.75 Hz frame rate by WormLab System (MBF Bioscience). Average speed and tracks were generated for each animal using WormLab software. To confirm the repeatability of the data, the locomotion speed was measured in two independent experiments in two days. For each mutant, around 10-40 animals were analyzed in one experiment. Significance was tested for each experiment."

4) In Figure 6A-D, it would be helpful to show data for null alleles for both lin-2 and frm-3, or show both presynaptic markers for a single allele, either lin-2 or frm-3. More consistent presentation of data in this portion of Figure 6 would be helpful for readers.

We used the same presynaptic marker (UNC-57::mCherry) in all indicated strains (wild type, *lin-2*$^{null}$, and *frm-3*$^{null}$) to keep the consistency of the data.

5) In Figure 5A, the reviewer finds the annotation excessive (arrows, green circle, red circle, purple circle). This makes it hard for the reader to properly see the presynaptic ultrastructure. The reviewer suggests substantially reducing annotations here.

We have removed the annotation for docked SVs and dense projection.

6) In Figure 1A and C, annotating the syb1036 and syb1019 alleles for base pair changes that lead to stop codons would be helpful.

Base pair changes have been added to Figure 1, as suggested.

7) Would be helpful if the legend for Figure 1 noted how speed was computationally tracked/analyzed.

Description has been added in Figure 1 legend.

**Reviewer #2:** This paper reports how C. elegans scaffolding proteins LIN-2 (a CASK homolog) and FRM-3 (a FARP homolog) regulate the function of NMJ synapses. C. elegans NMJ synapses are cholinergic. The authors make an argument in the Abstract of the paper that little is known about how scaffolds affect the development and function of cholinergic synapses. This is in contrast to information known about other excitatory and inhibitory synapses like glutamatergic or GABAergic synapses. By this framing, a characterization of LIN-2 and FRM-3 should expand our knowledge about cholinergic synapses and neurotransmission.

The first findings the authors report are behavioral and electrophysiological. Loss of either the lin-2 or frm-3 genes impairs locomotion and NMJ transmission. Double mutant combinations seem no more severe than single mutant combinations, suggesting a shared process or pathway for LIN-2 and FRM-3. Collectively, the authors report:

• behavior/locomotion data
• electrophysiological characterizations
• new null alleles that they generated for this study
• airtight genetic data localizing the electrophysiological functions to the muscle
• clear effects on acetylcholine receptors by imaging (suggesting impaired clustering)
• EM and fluorescence microscopy, suggesting that synapse development is normal
• meaningful distinctions between the cholinergic synapse roles and the GABAergic roles of these proteins
• structure and function and binding experiments to extend the above, suggesting importance for the PDZ domain of LIN-2 and the FERM domain of FRM-3 for binding – and specific domains of LIN-2 for function (PDZ and SH3)

Taking the data and results together, the authors have assembled an impressive and convincing dataset demonstrating that postsynaptic LIN-2 and FRM-3 are important for NMJ function. In this reviewer's opinion, this paper should interest many readers of PLOS Genetics, including synaptic physiologists and neuroscientists interested in synapse development. The authors generated a lot of valuable reagents for the field, especially new null alleles which shed light upon the functions of LIN-2 and FRM-3.

There are a few points that could be cleared up with text revisions.

Main Points

1. In terms of framing, there is a disconnect on what is known from prior work. The Abstract states, "far less is known about how scaffolds shape cholinergic synapses." Similarly, an early point of the Introduction reads, "much less is known about how nicotinic receptors are localized to synapses." But then immediately after that, the authors correctly list a whole panoply of factors known to stabilize AChR synapses (sentences that span pp. 3-4). This reads in an incongruous way.

We thank the reviewer for this suggestion. In the revised introduction, we no longer suggest that less is known about ACh synapses. Instead, we focus on the general interest in identifying scaffolds for post-synaptic receptors of all types:

Page 2:

"Changes in neurotransmitter receptor abundance at post-synaptic elements play a pivotal role in regulating synaptic strength. For this reason, there is significant interest in identifying and characterizing the scaffolds required for receptor localization at different synapses. Here we analyze the role of two *C. elegans* post-synaptic scaffolding proteins (LIN-2/CASK and FRM-3/FARP) at cholinergic neuromuscular junctions. Constitutive knockouts or muscle specific inactivation of *lin-2* and *frm-3* dramatically reduced spontaneous and evoked post-synaptic currents. These synaptic defects resulted from decreased abundance of two classes of post-synaptic ionotropic acetylcholine receptors (ACR-16/CHRNA7 and levamisole-activated AChRs). LIN-2's AChR scaffolding function is mediated by its SH3 and PDZ domains, which interact with AChRs and FRM-3/FARP. Thus, our findings show that post-synaptic LIN-2/FRM-3 complexes promote cholinergic synaptic transmission by recruiting AChRs to post-synaptic elements."

Page 4:

"To become functional, assembled ionotropic receptors must be transported to the plasma membrane and clustered at post-synaptic elements, where they are immobilized by synaptic scaffolding proteins. In rodents, several scaffolding proteins have been identified for localizing ionotropic GABA (neuroligin 2, collybistin, gephyrin), glutamate (PSD95, neuroligin 1 and 3), and ACh (rapsyn, MuSK) receptors. Analysis of invertebrate synapses have identified additional synaptic scaffolds. In *Drosophila*, ionotropic glutamate receptors are immobilized at neuromuscular junctions by CASK, Coracle/Band 4.1 proteins, Filamin, and Discs-Large (DLG) (Chen et al., 2005; Ataman et al., 2006; Chen and Featherstone, 2011; Lee and Schwarz, 2016). At *C. elegans* NMJs, ionotropic GABA receptors are localized by Neuroligin, FRM-3/FARP, LIN-2/CASK, and UNC-40/DCC (Tong, 2015; Maro, 2015; Tu, 2015; Zhou, 2020). Interestingly, a recent study suggests that *C. elegans* ionotropic ACh receptors (AChRs) are synaptically localized by an overlapping set of scaffolds (FRM-3/FARP, LIN-2/CASK, and UNC-40/DCC) (Zhou, 2021); however, this study did not analyze how cholinergic transmission and behavior were altered in these scaffold mutants."

LIN-2 and FRM-3 add to the body of knowledge, and their roles at the C. elegans NMJ are worth understanding. But as the text is currently written in the Introduction, it is unclear what major gaps in understanding are missing at cholinergic synapses in contrast to other types of synapses. It might be worth defining those gaps a little more clearly in the Introduction and then revisiting them in a Discussion section.

We have rephased the description of CASK in the introduction by adding major questions that we aim to address in this study, and discussion of our findings.

Page 6:

"Despite these advances in both vertebrates and invertebrates, key questions remain to be addressed regarding the function of CASK in cholinergic synapses. How does CASK and its binding partners regulate AChRs? Does CASK function by similar mechanisms at cholinergic and GABAergic synapses?"

Page 21:

"Knockout of either LIN-2A (*e1309* mutant) or both LIN-2A and LIN-2B causes decreased mEPSC amplitude without altering puffing ACh- or Levamisole-activated currents (Figure 1 and Figure 4), indicating normal AChRs trafficking and surface delivery. However, LIN-2A and LIN-2B appear to function differently in regulating $GABA_A$Rs. The decreased mIPSC amplitude but normal puffing GABA-activated currents in the *e1309* mutants (Tong et al., 2015), demonstrate that LIN-2A functions only for $GABA_A$Rs clustering and stabilization. In contrast, the *lin-2* null mutants exhibited a 50% reduction of puffing GABA-activated currents (Figure S2), indicating that the LIN-2B isoform is more likely involved in the delivery or retention of $GABA_A$Rs on the muscle surface. These results suggest that the two LIN-2 isoforms have different functions at cholinergic and GABAergic synapses."

2. There is a suggestion that loss of lin-2 and/or frm-3 unveils a trans-synaptic retrograde pathway that controls presynaptic release of acetylcholine. The authors mention such a pathway

very briefly in the Introduction and the Results. After that, the authors devote a very large section of the Discussion to retrograde signaling, and they draw parallels to other modalities previously described.

As detailed below, we agree that our data do not provide strong evidence for retrograde pre-synaptic effects of muscle LIN-2 and FRM-3. Consequently, we removed description of a potential retrograde signal from the results and discussion.

Based on the data, this reviewer understands why the authors are inferring a retrograde pathway. However, it seems like there are other interpretations of the data. Those alternate possibilities should be considered and mentioned alongside the comments about retrograde signaling in all parts of the manuscript where it appears.

The authors infer that retrograde signaling is occurring because postsynaptic loss of either lin-2 or frm-3 results in decreased quantal content, as well as decreased spontaneous mini frequency. The implication is that the loss of postsynaptic scaffolds somehow changes presynaptic release probability of acetylcholine. This is quite possible. But it also possible that the decreased quantal content and mini frequency are entirely due to disrupted ACh receptor organization. This sort of result has been observed at many model synapses, including NMJs. Therefore, diminishment of neurotransmitter receptor clusters could result in observed decreases in quantal content as well as decreases in mini frequency.

Alternatively, could it be the case that the miniature amplitudes are so small in the mutants that some are not so easily discerned? This would be consistent with the decreased frequency. If the real mini amplitude were smaller in the mutants than reported, then the estimated quantal content might not actually be diminished, and the retrograde signaling model would not necessarily apply for that scenario.

We thank the reviewer for this comment. As you suggest, the observed changes in mEPSC rate and evoked EPSC Quantal Content could be entirely explained by the dramatic reduction in post-synaptic ACh receptors in *frm-3* and *lin-2* mutants. This is now stated explicitly in the revised text:

Page 8:
"Thus, the decreased mEPSC rates observed in *lin-2* and *frm-3* mutants could be caused by the decreased mEPSC amplitude."

Prompted by this comment, we eliminated all discussion of potential retrograde pre-synaptic effects of muscle LIN-2 and FRM-3 in the revised text.

Minor Points

1. There is a small question about the conclusions drawn from the deletion experiments for LIN-2: are the authors certain that the versions of LIN-2 lacking either the PDZ or SH3 domains were still functional? If these domain deletions were to disrupt protein structure, then that might create a null condition. The observed results would be the same, but the reason would not be domain-specific.

Although the cholinergic synaptic transmission defects (mEPSCs, evoked EPSCs) in *lin-2* ΔSH3 and ΔPDZ mutants are similar to that in the null mutants, our recent analysis in GABAergic transmission showed that the mIPSC frequency was not changed in these mutants (Figure 8), different from the null mutant. These results clearly indicate that the LIN-2 proteins lacking SH3 or PDZ are still functional. The data has been added into the revised manuscript.

Page 17:

"The functional importance of the SH3 and PDZ domains was also evaluated in GABAergic synapses. The mIPSC amplitude in ΔSH3 and ΔPDZ mutants was decreased to a similar extent to that in *lin-2* null mutants (Figure 8G, H). However, the mIPSC frequency was not changed in the ΔSH3 and ΔPDZ mutants, unlike *lin-2* null mutants (Figure 2). These results suggest that the mutant LIN-2 proteins are expressed and retain some function."

2. The authors should be congratulated for generating and characterizing new null alleles. This is not a trivial task, and it offers a chance to clear up prior studies (for example, where frm-3(gk585) was referred to as a null).

We believe the strains we generated in this study will benefit future research in other labs.

The new nulls are used effectively to argue (via phenotypic non-additivity) that FRM-3 and LIN-2 are operating in the same pathway. One question for this reviewer – was it surprising that the double hypomorphic conditions did not show additivity? Unlike the nulls, it should be possible to enhance phenotypes of hypomorphs.

We agree with the reviewer that the lack of additivity in the double hypomorph is potentially interesting. This may indicate that the residual LIN-2 in *e1309* is insensitive to changes in FRM-3, or vice versa.

**Reviewer #3:** CASK (encoded by lin2) is a MAGUK family scaffold protein with CaM, PDZ, SH3 and GK domains. FARP (encoded by frm3) is a protein containing FERM, RhoGEF, and PH domains. Both have been implicated in synapse formation. Li et al. focused on two mutants lin2(e1309) and frm3(gk585) of C. elegans. The lin-2(e1309) mutation deletes an N-terminal region of LIN-2A but has no effect on LIN-2B whereas the gk585 allele is a deletion disrupting both frm-3a and b. Phenotypes of these two mutations on cholinergic and GABAergic NMJs were described. First, they authors showed that the mutants displayed locomotor deficits. Second, both mEPSC amplitude and frequency were reduced. Double mutants showed similar defects to those in single mutants. mIPSC frequencies were unaltered in lin2(e1309) and frm3(gk585) mutants but were dramatically decreased in null mutants (lin2null or frm3null). The authors selectively deleted the genes in motor neurons or muscles, and observed impaired mEPSCs and evoked EPSCs only by muscle-specific deletion. They also observed reduced abundance of synaptic AChRs, but not overall expression in muscles, in null mutant animals. They further demonstrated that lin2 directly binds to the AChR through its SH3 domain, which requires the interaction between lin2 and frn3 at the PDZ-FERM domains.

Because the frequency of EPSCs was reduced by muscle-specific mutation, but not motor neuron-selective mutation, the authors proposed a model of retrograde signaling by *lin-2* and *frm-3*.

As detailed above (see response to Review 2 major comment #2), we eliminated discussion of a potential retrograde signal in the revised text.

The Zhou 2021 paper also demonstrated the synergistic interaction of lin-2 and frm-3. Thus, the novelty here was questioned.

Our original submission did not clearly state how our results go beyond those described by Zhou 2021. Consequently, we understand the reviewer's concern that our results lack novelty. To address this concern, we systematically compare our results to those in the two prior papers from the Bessereau lab (Zhou et al. JCB 2021 and Nat. Comm. 2020):

1) We show that ACR-16/CHRNA7 and UNC-29 containing levamisole-sensitive ACh receptor (L-AChR) muscle puncta intensities are significantly reduced in both *lin-2* and *frm-3* mutants and additive defects were not observed in double mutants (Figure 4A-D). Zhou (2021) also analyzed ACR-16 puncta intensities in single mutants but did not analyze double mutants, nor did they analyze L-AChR levels.
2) We show that mEPSC amplitude, mEPSC rate, and evoked EPSC amplitudes are dramatically decreased in both *lin-2* and *frm-3* mutants (Figure 2). These recordings independently confirm that ACR-16 and UNC-29 receptor levels are reduced in these mutants. Zhou (2021) did not report any electrophysiological recordings in these mutants; consequently, their conclusions about ACh receptor levels were based solely on puncta analysis and were not confirmed by functional studies (i.e. electrophysiology).
3) We show that locomotion rate is modestly (but significantly) reduced in *lin-2* and *frm-3* mutants (Figure 1F), consistent with the residual synaptic UNC-29 localization in these mutants (Figure 4C, D). Zhou (2021) did not analyze locomotion rate in these mutants.

4) We show that mEPSC decay kinetics are significantly slower in *lin-2* and *frm-3* mutants (Figure S5). These results suggest that UNC-29 containing ACh receptors (which have slower intrinsic inactivation kinetics) contribute more to residual synaptic currents in these mutants. Thus, these results also suggest that FRM-3 and LIN-2 have a stronger effect on ACR-16 synaptic levels than on UNC-29 levels. Zhou (2021) did not record mEPSCs in these mutants.
5) We show that pre-synaptic ultrastructure, and the abundance of pre-synaptic markers (e.g. UNC-57) are not significantly disrupted in *lin-2* and *frm-3* mutants, suggesting that defects in these mutants are primarily post-synaptic. Zhou did not analyze ultrastructure or pre-synaptic markers in these mutants.
6) We show that mIPSC amplitudes and rates are significantly reduced in *lin-2* and *frm-3* null mutants (Figure 2C, D), consistent with decreased synaptic $GABA_AR$ levels in these mutants as shown in our earlier paper (Tong 2015, eLife). Zhou (2020) showed that evoked IPSCs were reduced in these mutants, but did not analyze mIPSCs. Thus, Zhou (2020) could not distinguish pre- and post-synaptic defects in GABAergic transmission.
7) We show that LIN-2 interacts with both ACR-16 and UNC-29 (Figure 7). Zhou (2021) only tested the interaction between LIN-2 and ACR-16 but not UNC-29.

For these reasons, we hope that the reviewers will agree that our results significantly strengthen the conclusion that FRM-3 and LIN-2 recruit ACR-16/CHRNA7 and UNC-29/L-AChR receptors to post-synaptic elements in muscles, and greatly extend our understanding or how these scaffolds regulate cholinergic transmission.

Prompted by this comment, we revised the text to make sure that the Zhou 2020 and 2021 papers have been accurately cited.

Second, the paper overall seemed to be descriptive, without much mechanistic insight on differential regulation on cholinergic versus GABA synapses and on molecular nature of retrograde signaling. The authors have much cleaner mutants (null and muscle- and motor neuron-specific cKOs), but did not carefully characterize those. The authors suggest that presynaptic and locomotion deficits were due to postsynaptic loss of lin2 and frm3, but clean muscle-specific mutants were not systematically for locomotion, AChR abundance, morphology of presynaptic terminals, and the number and priming of synaptic vesicles. The authors have the lines and tools and should provide such results.

As requested, we have done more behavioural and electrophysiological analysis in *lin-2* and *frm-3* $Muscle^{Cre}$ mutants, including kinetics of averaged minis, surface AChR and $GABA_AR$ measurement (by puff), and locomotion speed.  The requested receptor imaging and EM experiments were not conducted because: 1) the $Muscle^{Cre}$ mutants exhibit nearly the same defects in mEPSC and mIPSC amplitude; therefore, there is little doubt that AChRs and $GABA_ARs$ are also decreased in $Muscle^{Cre}$ mutants; and 2) synaptic ultrastructure is normal in *lin-2* and *frm-3* null mutants; consequently, $Muscle^{Cre}$ mutants are unlikely to exhibit ultrastructural defects.

Our new analysis showed that: 1) *lin-2* and *frm-3* $Muscle^{Cre}$ mutants exhibit significantly slower mEPSC decay than control animals, consistent with *lin-2* and *frm-3* null mutants. This likely results from a greater contribution of L-AChRs, which mediate synaptic currents with

smaller amplitude but slower inactivation. 2) ACh- and levamisole-activated currents (by puff) in *lin-2* and *frm-3* Muscle$^{Cre}$ mutants were indistinguishable from that in control animals and Neuron$^{Cre}$ mutants, demonstrating that LIN-2 and FRM-3 are not required for the trafficking of AChRs. By contrast, GABA-activated currents were normal in *frm-3* Muscle$^{Cre}$ mutants but significantly decreased in *lin-2* Muscle$^{Cre}$ mutants, compared to their controls and Neuron$^{Cre}$ mutants. These results are consistent with the observations in the null mutants of *lin-2* and *frm-3*, further demonstrating that the mechanisms that LIN-2 regulates AChRs and $GABA_A$Rs are different. 3) locomotion speed was significantly decreased in the *lin-2* Muscle$^{Cre}$ mutants, but not the Neuron$^{Cre}$ mutants. However, we did not observe a locomotion decrease in *frm-3* Muscle$^{Cre}$ and Neuron$^{Cre}$ mutants. One possibility is that the efficiency of the Cre recombinase inactivating *frm-3* is not as strong as that for *lin-2*. This is likely as the mIPSC frequency is significantly decreased in *lin-2* Muscle$^{Cre}$ mutants, but not changed in *frm-3* Muscle$^{Cre}$ mutants (Figure 3). These results have been incorporated into the revised manuscript.

Third, it was unclear why lin2(e1309) and frm3(gk585) were focused, which miss a particular domain or disrupt different isoforms. However, whether these domains/isoforms are involved were not certain. A reasonable experiment was to determine which phenotypes in muscle-specific cKO could be rescued by lin2(e1309) and frm3(gk585) mutants.

The *lin-2(e1309)* and *frm-3(gk585)* mutants were used in several prior studies to show how LIN-2 and FRM-3 regulate synaptic function, but they are not the focus of this paper. These two mutants were used in this study just to clarify that although they exhibit substantial decrease in cholinergic and GABAergic synaptic transmission, they are not null mutants. By contrast, the null mutants generated in this research exhibit not only stronger phenotype, but also previously unknown functions of LIN-2 and FRM-3 in both cholinergic and GABAergic synapses. We hope that reviewers will agree that investigating the function of different LIN-2 and FRM-3 isoforms and protein domains can be addressed in future studies.

Fourth, the paper seemed to focus on "development" of synapses, but failed to present a time-course of different phenotypes. For example, which deficits (pre versus post-synaptic) occurs earlier, when?

In this study, we did not investigate the functions of LIN-2 and FRM-3 during development. In fact, all phenotypes, including behaviour, synaptic transmission, and synaptic imaging, were examined in adult stage. It is extremely difficult to perform electrophysiology in younger stages (L1-L4) in worm. We therefore cannot present a time course of both presynaptic and postsynaptic defects. However, we'd like to test this in the future when we can conduct recordings more stably at larval stages.

Fifth, the authors stated that "Detection of spontaneous mEPSCs is impaired when their amplitudes are decreased; consequently, the decreased mEPSC frequency in lin-2(e1309) and frm-3(gk585) mutants could be caused by the smaller mEPSC amplitude." By the authors' argument, the frequency reduction must result from specific loss of low-amplitude mEPSCs. Was this the case? It would be important to determine whether frequency reduction did not result from a preferred reduction of low-amplitude mEPSCs.

Yes, decrease in mEPSC frequency can be caused by decrease in mEPSC amplitude. This has been observed in many synapse types. We have added the results that mEPSC frequency is reduced by 50% in *acr-16* mutants, in which mEPSC amplitude is largely decreased. As detailed above, because post-synaptic currents are so dramatically reduced in *lin-2* and *frm-3* mutants we are unable to use these currents as an assay for pre-synaptic SV fusion. For this reason, we no longer speculate about retrograde pre-synaptic defects in these mutants.

Page 8:

"The decreased mEPSC amplitude suggests that postsynaptic ionotropic AChRs are disrupted in *lin-2* and *frm-3* mutants (e.g., decrease in synaptic abundance). It should be noted that smaller mEPSC amplitudes may cause some mEPSC events to become undetectable, thereby reducing mEPSC frequency. This is supported by the fact that mEPSC frequency is decreased by 55% in *acr-16* mutants, which lack nicotine-sensitive receptors that are orthologous to mammalian CHRNA7 receptors (Figure S2A, B). Thus, the decreased mEPSC rates observed in *lin-2* and *frm-3* mutants could be caused by the decreased mEPSC amplitude."

Sixth, the roles of lin-2 and frm-3 in the GABAergic synapses are poorly understood. Apparently, the authors have a niche here to explore this deeply. Why e1309 and gk585 mutants altered cholinergic synapses, but had no effect on the frequency of mIPSCs of GABAergic synapses? Does this suggest that GABAergic synaptic defects are secondary? How about domains or isoforms that were specifically impacted by these mutations? Were there GABAergic phenotypes (such as IPSC) in lin-2 and frm-3 Neuron- and Muscle-Cre cKOs? A careful time-course analysis of the types of synapses and rescue, as described above, would be informative. Relatedly, were there any deficits at GABAergic synapses in delete-SH3 and/or delete-PDZ mutants?

Differential changes in cholinergic and GABAergic transmission have actually been observed in many other mutants. Although the mechanisms are still unclear, it appears that spontaneous GABA release is relatively easier to occur (Liu et al., 2018, Cell Reports). As shown in Figure 1, in *lin-2(e1309)* only the LIN-2a isoform is disrupted but the LIN-2b isoform is not affected, and in *frm-3(gk585)* both FRM-3 a and b isoforms are affected. One possibility for the unaltered mIPSC frequency in *lin-2(e1309)* and *frm-3(gk585)* mutants is that the LIN-2b isoform and the remaining activity of FRM-3 are sufficient to mediate spontaneous GABA release. It is unlikely a secondary result, as we have previously demonstrated that an acute block of ACh release at the NMJ does not alter GABA release (data not shown). As requested, spontaneous GABA release was examined in *lin-2* and *frm-3* muscle KO mutants, and *lin-2* ΔSH3 and ΔPDZ mutants. We found that the mIPSC amplitude is dramatically decreased in these mutants, whereas the mIPSC frequency was only decreased in *lin-2* muscle KO mutants but not other mutants. Also, our data support that the truncated LIN-2 lacking SH3 or PDZ is still able to mediate spontaneous GABA release, suggesting different mechanisms of LIN-2 in mediating ACh and GABA release. We have added these results in the revised manuscript.

Page 9-10:

"Similarly, Muscle$^{Cre}$ also significantly decreased the mIPSC amplitude in *lin-2(nu743)* and *frm-3(nu751)* mutants (Figure 3B, C, F, G), indicating that LIN-2 and FRM-3 also act in muscle to regulate postsynaptic $GABA_A$ receptors, consistent with prior studies (Tong et al., 2015; Zhou et al., 2020). However, unlike cholinergic synapses, Muscle$^{Cre}$ decreased the mIPSC frequency by only 50% in *lin-2(nu743)* mutants (90% in *lin-2* null mutants), and did not change mIPSC frequency in *frm-3(nu751)* mutants. Collectively, these results indicate that LIN-2 and FRM-3 control synaptic transmission at both cholinergic and GABAergic synapses but that the detailed mechanisms likely differ at these two synapses."

Page 17:

"The functional importance of the SH3 and PDZ domains was also evaluated in GABAergic synapses. The mIPSC amplitude in ΔSH3 and ΔPDZ mutants was decreased to a similar extent to that in *lin-2* null mutants (Figure 8G, H). However, the mIPSC frequency was not changed in the ΔSH3 and ΔPDZ mutants, unlike the *lin-2* null mutants (Figure 2). These results suggest that the mutant LIN-2 proteins are expressed and retain some function."

Other concerns

More credits should be given to two papers by Zhou et al. in the introduction. The statement of "…, the functions of CASK and its binding proteins at cholinergic synapses have yet to be explored" seemed to be inappropriate.
Regarding retrograde mechanisms, the hypothesis of the neurexin-neuroligin pathway needs more discussion. Neuroligin may be involved in the recruitment of $GABA_AR$ but not that of AChR in *C. elegans*. In fact, syndecan (rather than neuroligin) was suggested to play the role in the cholinergic synapses (Zhou et al., J. Cell Biol. 2021).

We have made changes regarding the inappropriate sentence in the introduction of the revised manuscript. The Zhou's paper has been cited.

Page 5:

"In *C. elegans*, LIN-2/CASK together with its binding partner FRM-3 (ortholog of the mammalian scaffold FARP), promote GABAergic synaptic transmission by immobilizing $GABA_A$ receptors at the NMJ (Tong et al., 2015; Zhou et al., 2020). A recent study from Zhou et al has revealed that LIN-2/CASK and FRM-3/FARP are also required for stabilizing acetylcholine receptors at the C. elegans NMJ (Zhou et al., 2021)."

As we have removed the results of the retrograde pathway in the revised manuscript, we did not discuss the potential involvement of syndecan.

Discussion could be improved by comparing worm NMJs with those in mammals, in particular for conserved proteins such as rapsyn.

A paragraph has been added in the discussion section.

Page 17:

"Different from the NMJs in mouse and fly which use ACh or glutamate as their primary neurotransmitters, the *C. elegans* NMJs contain both cholinergic and GABAergic synapses which release ACh and GABA as two primary neurotransmitters to regulate muscle activity (Richmond and Jorgensen, 1999). This allows us to use the worm NMJ investigate three different neurotransmitter receptors, nAChRs, L-AChRs, and $GABA_ARs$. Moreover, the major known synaptic organizers found in vertebrates, such as PSD-95, rapsyn, MuSK, and neuroligin, also exist in worm NMJ, suggesting that the regulatory mechanisms of the AChRs and $GABA_ARs$ in *C. elegans* are similar to those in other species."

Figure 5 panel A, the value of the y-axis is inconsistent with the scale bar, the unit of the y-axis should be micrometer square rather than millimeter square? In panel B, the quantification data showed a comparable mean value between the three groups; however, the representative images showed obviously higher numbers of SVs in the mutant groups than that in the control group.

The unit of the y-axis has been corrected. A new control image was used in panel A.

The 1019 and 1036 strains were referred to as “null mutants” at some places. This inconsistency should be avoided, both in the Results and Figures.

We have now used *lin-2*$^{null}$ and *frm-3*$^{null}$ throughout the text and figures.

Figure 2, panels A-D, for the reduction of amplitude of PSCs, representative traces showed much more dramatic changes than the quantitative data. Same issue in Figure 3, panels B-C and Figure 8B, C.

We have used new representative traces in the revised manuscript.

Figure 4, panels A,B, representative images showed much more dramatic reduction than the quantitative data.

New representative images have been chosen to reflect the reduction more accurately.

Figure 6, panel E, “syb1018” should be “syb1019”.

This has been corrected.

A graphic elucidation for the interaction between lin2, frm3, and AChRs and the related domains should be helpful.

A model figure (Figure 9) has been added in the revised manuscript.
