# Supplementary material for: CASK and FARP localize two classes of post-synaptic ACh receptors thereby promoting cholinergic transmission
Source: PLoS Genet. 2022 Oct 24;18(10):e1010211. doi: 10.1371/journal.pgen.1010211 (PMC9632837; doi:10.1371/journal.pgen.1010211)
Supplement: Attachment — Submitted filename: Grill first review.docx [file pgen.1010211.s012.docx]

This study by Hu and colleagues aims to understand how LIN-2/Cask and FRM-3/FARP regulate cholinergic transmission. While the LIN-2/FRM-3 complex has been evaluated in GABA transmission, this study examines effects on cholinergic transmission for the first time. A highlight of the study is the tremendous range of approaches including behavioral analysis, extensive electrophysiology, traditional genetics, cell-specific knockouts, yeast two-hybrid and CRISPR editing.

The study takes two important mechanistic steps. 1) LIN-2 and FRM-3 are required to localize acetylcholine receptors to postsynaptic terminals. This provides a cell biological explanation for the cholinergic transmission defects in lin-2 and frm-3 mutants. 2) The SH3 domain of LIN-2 was found to bind ACR-16 and UNC-29 Ach receptor subunits, and the LIN-2 PDZ domain interacts with FRM-3. Results with CRISPR editing to remove specific domains in LIN-2 and functional outcomes were consistent with biochemical findings. Thus, the authors’ results suggest that the FRM-3/LIN-2 complex binds UNC-16 and UNC-29 Ach receptors to regulate their localization and cholinergic transmission.

Below the reviewer raises several concerns with the goal of solidifying what is a potentially excellent study.

**Major Comments**

1) For Figures 1 and 2, rescue experiments for frm-3 and lin-2 single mutants, and analysis of lin-2 frm-3 double mutants using null alleles are needed to strengthen conclusions. Given that the authors argue LIN-2 and FRM-3 form a complex and function in the same pathway, multi-gene genetics needs to be done with null alleles to be conclusive.

Regarding electrophysiology it seems reasonable to only perform rescues and double mutant analysis on a portion of the cholinergic transmission readouts.

2) LIN-2 and FRM-3 affect presynaptic and postsynaptic transmission. Isn’t it important to test their cell-specific activity in not only postsynaptic muscles but also presynaptic cholinergic motor neurons? While the authors have used a pan-neuronal driver this is not necessarily specific enough and might not yield efficient CRE recombination in cholinergic motor neurons. This is an important experiment to shore up the trans-synaptic signaling model because LIN-2 and FRM-3 are expressed in cholinergic motor neurons.

3) The authors do a nice job discussing trans-synaptic effects of MAGUKs, Mint and FARP. While the reviewer very much appreciates this, a question comes to mind. Why would frm-3 and lin-2 principally function postsynaptically in muscle but yield stronger effects on presynaptic transmission (mEPSC frequency) than postsynaptic transmission (EPSC amplitude)? Is this the case in the other examples the authors cite?

4) Is the evoked EPSC as strong with muscle specific knockouts as null alleles? This does not look to be the case. Could the authors clarify?

5) The use of CRISPR to delete SH3 and PDZ domains from LIN-2 is a very nice addition to the study, and complementary to yeast two-hybrid results. However, the reviewer urges some caution in interpreting the delta SH3 and delta PDZ CRISPR alleles. Are the authors sure that these deletion mutants do not destabilize the overall LIN-2 protein structure creating null alleles? Similarly, negative results in yeast two hybrid experiments might result from failure of construct expression.

**Minor Comments**

1) The reviewer was a bit puzzled. What is the role of lin-2 and frm-3 in motor neurons, if it is acting exclusively in muscle to affect both pre and postsynaptic cholinergic transmission? Is anything know about other roles for LIN-2 or FRM-3 in presynaptic neurons/terminals? Perhaps the authors might comment on this in the Discussion?

2) Wouldn’t such large effects on cholinergic transmission, as observed in lin-2 and frm-3 null mutants, precipitate larger defects in locomotion? Perhaps another known control for reduced cholinergic transmission might be included in locomotor behavioral studies?

3)The data in Figure 1F seems to be presented as individual animals. It would be helpful if the legend noted the number of independent experiments that were performed. The reviewer is concerned that reporting individual animals is not ideal compared to reporting the average locomotion speed from multiple experiments and then running statistics. The authors might consider reanalyzing this data. C. elegans is a model where large numbers of animals can be analyzed and data can be presented across independent experiments rather than using individual animals.

4) In Figure 6A-D, it would be helpful to show data for null alleles for both lin-2 and frm-3, or show both presynaptic markers for a single allele, either lin-2 or frm-3. More consistent presentation of data in this portion of Figure 6 would be helpful for readers.

5) In Figure 5A, the reviewer finds the annotation excessive (arrows, green circle, red circle, purple circle). This makes it hard for the reader to properly see the presynaptic ultrastructure. The reviewer suggests substantially reducing annotations here.

6) In Figure 1A and C, annotating the syb1036 and syb1019 alleles for base pair changes that lead to stop codons would be helpful.

7) Would be helpful if the legend for Figure 1 noted how speed was computationally tracked/analyzed.
